# Detection of differentially culturable tubercle bacteria in sputum from drug-resistant tuberculosis patients

**DOI:** 10.3389/fcimb.2022.949370

**Published:** 2022-09-09

**Authors:** Bhavna G. Gordhan, Astika Sewcharran, Marothi Letsoalo, Thilgavathy Chinappa, Nonhlanhla Yende-Zuma, Nesri Padayatchi, Kogieleum Naidoo, Bavesh D. Kana

**Affiliations:** ^1^ Department of Science and Technology/National Research Foundation Centre of Excellence for Biomedical Tuberculosis (TB) Research, School of Pathology, Faculty of Health Sciences, University of the Witwatersrand and the National Health Laboratory Service, Johannesburg, South Africa; ^2^ Centre for the AIDS Programme of Research in South Africa (CAPRISA), University of KwaZulu-Natal, Durban, South Africa; ^3^ South African Medical Research Council (SAMRC)-Centre for the AIDS Programme of Research in South Africa (CAPRISA) Human Immunodeficiency Virus- Tuberculosis (HIV-TB) Pathogenesis and Treatment Research Unit, University of KwaZulu-Natal, Durban, South Africa

**Keywords:** tuberculosis, drug resistance, differentially culturable tubercle bacteria (DCTB), culture filtrate (CF), resuscitation promoting factors (Rpfs)

## Abstract

Several studies described the presence of non-replicating, drug-tolerant differentially culturable tubercle bacteria (DCTB) in sputum from patients with active tuberculosis (TB). These organisms are unable to form colonies on agar but can be recovered in liquid media supplemented with culture filtrate as a source of growth factors. Herein, we undertook to investigate the response of DCTB during the treatment of individuals with drug-resistant TB. A cohort of 100 participants diagnosed with rifampicin-resistant TB were enrolled and prospectively followed to monitor response to therapy using routine culture and limiting dilution assays, supplemented with culture filtrate (CF) to quantify DCTB. Fifteen participants were excluded due to contamination, and of the remaining 85 participants, 29, 49, and 7 were infected with rifampicin mono-resistant (RMR), multidrug-resistant (MDR), or extremely drug-resistant (XDR) TB, respectively. Analysis of baseline sputum demonstrated that CF supplementation of limiting dilution assays detected notable amounts of DCTB. Prevalence of DCTB was not influenced by smear status or mycobacterial growth indicator tube time to positivity. CF devoid of resuscitation promoting factors (Rpfs) yielded a greater amount of DCTB in sputum from participants with MDR-TB compared with those with RMR-TB. A similar effect was noted in DCTB assays without CF supplementation, suggesting that CF is dispensable for the detection of DCTB from drug-resistant strains. The HIV status of participants, and CD4 count, did not affect the amount of DCTB recovered. During treatment with second-line drug regimens, the probability of detecting DCTB from sputum specimens in liquid media with or without CF was higher compared with colony forming units, with DCTB detected up to 16 weeks post treatment. Collectively, these data point to differences in the ability of drug-resistant strains to respond to CF and Rpfs. Our findings demonstrate the possible utility of DCTB assays to diagnose and monitor treatment response for drug-resistant TB, particularly in immune compromised individuals with low CD4 counts.

## Introduction

Drug-resistant tuberculosis (DR-TB) is a global health crises, with 206,030 people notified with multidrug-resistant or rifampicin mono-resistant TB (MDR/RMR-TB) in 2019, a 10% increase from 186,883 in 2018 (World Health Organization, 2020). MDR *Mycobacterium tuberculosis* strains display resistance to the first-line drugs rifampicin and isoniazid, while pre-extremely drug-resistant (pre-XDR) strains display additional resistance to fluoroquinolones or injectables. XDR strains are MDR with additional resistance to fluoroquinolones and injectables. Further resistance to drugs used to treat XDR-TB results in totally drug-resistant (TDR) strains that are often programmatically untreatable (Dheda et al., 2017). The high mortality associated with drug-resistant TB, often driven by HIV coinfection in certain endemic countries, represents a growing public health threat. Although new drugs such as bedaquiline and delamanid, as well as repurposed drugs such as linezolid and clofazimine, are available for treatment of DR-TB, limited access to these agents and/or the inability to generate an effective combinatorial regimen drives poor outcomes. The high cost, long treatment duration (18–24 months), and debilitating or life-threatening toxicity of second-line drugs pose further challenges in the management of DR-TB (Dookie et al., 2018).

Despite widespread rollout of molecular diagnostics for TB, early detection of drug resistance is limited to rifampicin resistance only (Boehme et al., 2010). The current gold standard for testing *M. tuberculosis* drug susceptibility (DST) is culture-based, which is protracted and delays appropriate linkage to care. This can lead to inappropriate prescription of drugs with devastating side effects or underdosing leading to further progression of the disease and resistance (Schaberg et al., 1996; Melchionda et al., 2013). Hence, improving the efficiency of current culture-based diagnostic approaches is expected to have a beneficial effect on the diagnostics value chain. In this regard, detection of non-replicating, non-platable bacterial populations becomes paramount. Mathematical modeling of sequential bacteriological load data from clinical trials showed a negative correlation between mycobacterial growth indicator tube (MGIT) and colony forming units (CFU) on agar plates, suggesting the presence of a greater mycobacterial load in liquid than on solid medium (Bowness et al., 2015). Several studies demonstrated the presence of differentially culturable/detectable tubercle bacteria (DCTB/DDTB) in sputum that are unable to grow on agar plates but can be resuscitated in liquid media supplemented with culture filtrate (CF) as a source of growth stimulatory factors (Mukamolova et al., 2010; Chengalroyen et al., 2016; Dartois et al., 2016; Dusthackeer et al., 2019; Gordhan et al., 2021). The growth stimulatory effect of CF has been ascribed to the five resuscitating promoting factors (Rpfs) from *M. tuberculosis*, which display the ability to resuscitate non-culturable bacteria (Mukamolova et al., 2002; Kana et al., 2008; Mukamolova et al., 2010). However, sputum samples also harbor a population of Rpf-independent DCTB, which do not require Rpfs for growth, and CF-independent DCTB, which are able to spontaneously resuscitate in liquid media without CF (Chengalroyen et al., 2016). These data underscore the complexity of phenotypically diverse populations in sputum (Dartois et al., 2016).

Detection of DCTB facilitates diagnostic pickup of viable *M. tuberculosis* in patients with negative results by routine testing, particularly those with HIV coinfection (Dusthackeer et al., 2019; McIvor et al., 2021). In addition to Rpfs, cAMP and fatty acids were effective in the detection of non-culturable bacterial populations in a laboratory model; however, these compounds offered no benefit in the recovery of such organisms from sputum samples (Shleeva et al., 2013; Gordhan et al., 2021). DCTB also display drug tolerance, compared with replicating bacteria, suggesting that early TB treatment eliminates only the conventionally culturable sub-population and not the total *M. tuberculosis* bacillary load (Turapov et al., 2016; Saito et al., 2017). Supporting this hypothesis, DCTB were shown to increase significantly after 2 weeks of treatment with standard first-line drugs in HIV-negative Haitian subjects with drug-sensitive TB, suggesting that drug treatment reduces CFU and concurrently increases the proportion of DCTB (McAulay et al., 2018).

A recent comparative analysis for the presence of DCTB prior to and after treatment from participants with DR-TB revealed that sputum from 29% of subjects displayed DCTB prior to treatment initiation, which remained steady after 2 weeks of treatment with second-line drugs. However, after 2 months of treatment, the proportion of DCTB reduced to almost half in subjects with drug-susceptible TB, while no DCTB was detected in sputum from subjects with DR-TB (Zainabadi et al., 2021). These observations suggest that DCTB from DR *M. tuberculosis* strains may be more susceptible to clearance with second-line drugs. Herein, we undertook to further investigate this effect in DR strains as this has important implications for treatment duration. We also probed Rpf dependency of DCTB in DR *M. tuberculosis*. Our study was nested in a large randomized observational cohort of HIV-positive and -negative individuals from South Africa. The resulting data correlated with previous findings showing the presence of DCTB in the pretreatment sputum, the proportion of which was significantly influenced by the drug resistance status. Unlike our previous findings with drug-susceptible TB, DCTB levels did not correlate with the HIV status or CD4 counts of the participant. The longitudinal analysis assessed the effect of CF on the probability of detecting DCTB over treatment time compared with other standard readouts of bacterial load.

## Results

### Quantification of DCTB in sputum specimens from patients with DR-TB using CF from *M. tuberculosis*

**Figure 1:**
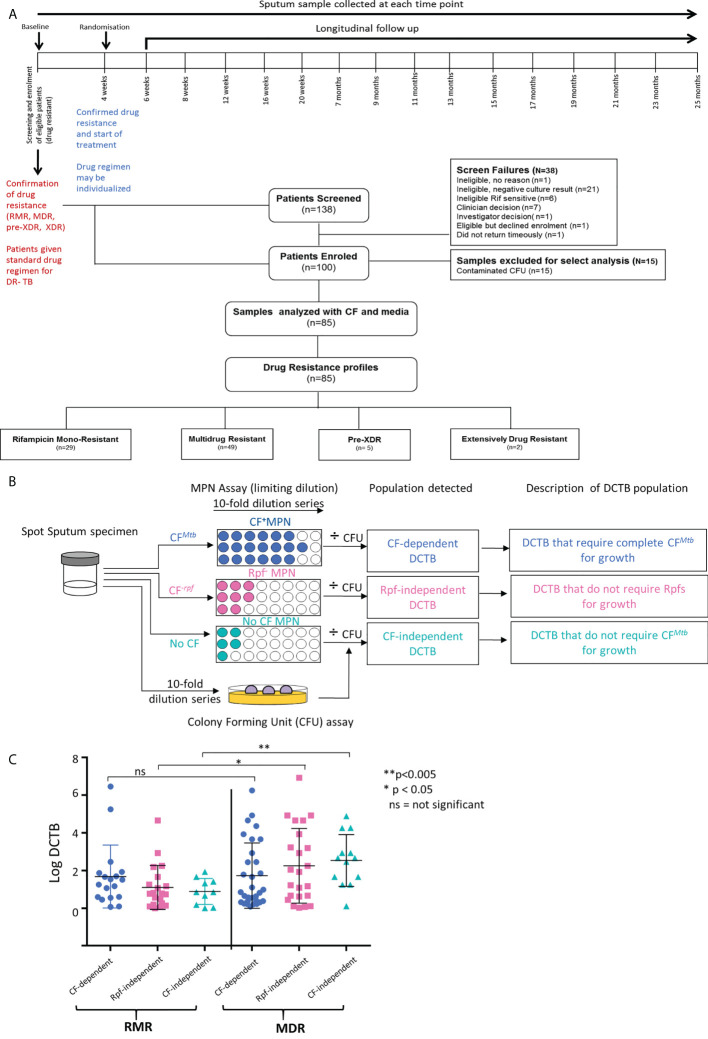
Participant disposition flow chart and assessment of DCTB in baseline sputum samples. **(A)** Study timeline and participant disposition flow chart for individuals recruited in this study. **(B)** Flow chart for DCTB assessment of sputum specimens in real time obtained at baseline (screening). Sputum samples were decontaminated and the resulting bacteria assessed by CFU and Most Probable Number (MPN) limiting dilution assays containing CF with and without Rpfs to detect DCTB. To control for the effect of CF in growth stimulation, fresh Middlebrook media was used (No CF MPN). DCTB count was obtained by dividing the MPN values (with or without CF) by CFU counts, the latter depicting conventionally culturable bacteria. **(C)** Median DCTB counts from different MPN assays and resistance categories (RMR, rifampicin mono-resistant; MDR, multidrug resistant). Values from specimens that had detectable levels of DCTB were used. Error bars depict the interquartile range. An unpaired t-test was used, where *p-value <0.05, **p-value <0.005 and ns, not significant.

The treatment timeline and participant disposition flow chart for the cohort used is shown in **Figure 1A**. This cohort was part of a primary study aimed at investigating the use of whole genome sequencing to direct regimen building. Eligible participants were recruited based on rifampicin resistance on GeneXpert at screening, and a sputum sample was taken from individuals who consented for inclusion. Participants initiated a standard DR-TB treatment regimen upon detection of rifampicin resistance, with regimen modification after drug susceptibility testing 4 weeks later (randomization). Intervention arm patients received an individualized DR-TB regimen based on whole genome sequence (WGS) results, but the primary outcome of this was not available at the time of this submission as the study is ongoing. Participants were followed longitudinally for 25 months (**Figure 1A**). We screened 138 individuals for DR-TB and enrolled 100. The large number of screen failures was due to negative culture results (n=21) and six participants were sensitive to rifampicin. Exclusion of seven individuals was based on the clinician’s decision while one individual declined enrolment. Exclusion of a further three individuals was based on the following criteria: one did not return timeously, the second was an investigator decision, and for the third, no reason for exclusion was recorded. A summary of baseline participant demographics and laboratory data is provided in **Table 1**. All included participants were sputum GeneXpert-positive with medium or high bacterial loads and a MGIT time to positivity of 7, 6, and 9 days for RMR, MDR, and XDR participants, respectively. A total of 29 participants displayed RMR, 49 were infected with MDR-TB, while 5 were infected with pre-XDR and 2 with XDR-TB. For demographics, we combined the pre-XDR and XDR categories in **Table 1**. In all three resistance groups, there were more men, and the median age of participants was 32 (RMR), 36 (MDR), and 31 (XDR) years. After exclusion of 15 participants, sputum at screening from the remaining 85 individuals was analyzed using the most probable number (MPN—to detect DCTB) assay and colony forming unit (CFU—to detect conventionally culturable bacteria) assessments as outlined in **Figure 1B** and described further in Supplementary Information. Of these, 7% (6/85) of the participants received anti-TB drugs prior to the baseline measure (the numbers of days of treatment were 1, 1, 1, 1, 11, and 16, with a median of 0 days of TB treatment for all 85 participants). The participants were stratified into three groups based on their drug resistance status; the percentage harboring DCTB in the RMR and MDR groups was equivalent (approximately, 70%–75%, **Supplementary Figure 1A**), while in the XDR group, the amount of DCTB was reduced to half and a greater proportion of participants displayed no DCTB. As previously reported (Chengalroyen et al., 2016), we were able to demonstrate that the CF from *M. tuberculosis* with or without Rpfs was necessary to recover the maximum amount of DCTB in sputum from patients infected with DR *M. tuberculosis* compared with specimens tested in media without CF (**Supplementary Figure 1B**). This was not seen in pre-XDR/XDR isolates, which was most likely due to the small sample size; hence, this group was excluded from subsequent analyses. Our analysis to assess the effect of CF on the recovery of DCTB included specimens wherein no DCTB were detected (MPN/CFU<1, **Supplementary Figure 1B**). This approach allowed for a comparative assessment of CF supplementation in the detection of DCTB. We next sought to determine if drug resistance status (RMR or MDR) affected the amount of DCTB recovered, and for this, we excluded data for all specimens with no DCTB. No differences in the recovery of DCTB between specimens containing RMR or MDR isolates were noted when complete CF (detecting CF-dependent DCTB) was used in MPN assays (**Figure 1C**). In contrast, the use of CF without Rpfs (detecting Rpf-independent DCTB) or complete exclusion of CF (detecting CF-independent DCTB) from MPN assays yielded more DCTB in specimens with MDR isolates (**Figure 1C**).

**Table 1:** Demographics and laboratory diagnostic data for study participants.

	Measurement		
	Rif-mono-resistant participants (n=29)	Multidrug-resistant participants (n=49)	Pre and extremely drug-resistant participants (n=7^§^)
**Variables (n=85)**			
**Demographics**			
Sex:			
Women (%)	14 (48)	17 (35)	1(14)
Men (%)	15 (52)	32 (65)	6 (86)
Age, yr, Median (IQR)	32 (28–39.5)	36 (29–43)	31 (24–34)
Weight, kg, Median (IQR)	59.5 (47.3–67.5)	57.5 (52.5–64.25)	55 (45.5–58.0)
Height, mm, Median (IQR)	167 (162.3–173.2)	167 (162.5–175)	168 (163.5–170)
BMI, Median (IQR)	19 (17.1–23.6)	20.10 (19.1–21.5)	19 (18.4–19.3)
**HIV Status, n (%)**			
Positive	22 (76)	32 (65)	4 (57)
Negative	7 (24)	17 (35)	3 (43)
CD4 Count*, median (IQR)	235.0 (146.5–334.0)	285.0 (149.3–376.8)	63.0 (19.0–130.0)
**Conventional TB diagnosis, n (%)**			
Smear grade negative	8 (28)	14 (29)	3 (43)
Smear grade positive	21(72)	35 (71)	4 (57)
**MGIT Time to Positivity, d, median (IQR)**	7.0 (6.0–13.5)	8.0 (5.0–11.75)	9.0 (8.0–13.0)
**Log Bacterial load by MPN assay#**			
CF-dependent MPN, log median (IQR)	4.29 (2.4–5.6)	3.93 (2.3–5.7)	3.66 (0.0–4.2)
Rpf-independent MPN, log median (IQR)	4.26 (2.3–5.6)	3.60 (1.7–5.3)	3.66 (0.0–4.1)
CF-independent MPN, log median (IQR)	1.66 (0.4–4.6)	1.66 (0.0–2.9)	1.66 (0.0–2.7)
CFU, log median (IQR)	3.85 (0.0–5.2)	3.21 (0.0–4.9)	2.11 (0.0–3.8)
**Amount of DCTB (MPN/CFU)#**			
CF-dependent DCTB, log median (IQR)	0.55 (0.0–1.6)	0.32 (0.0–1.7)	0.0 (0.0–1.9)
Rpf-independent DCTB, log median (IQR) CF-independent DCTB, log median (IQR)	0.27 (0.0–1.1) 0.0 (0.0–0.4)	0.0 (0.0–1.6) 0.0 (0.0–0.7)	0.0 (0.0–2.2) 0.0 (0.0–0.0)

Definition of abbreviations: yr, years; d, days; kg, kilograms; mm, millimeters; BMI, body mass index; IQR, interquartile range; MGIT, mycobacterial growth indicator tube.

*Only in people who are HIV infected.

^#^On screening specimen.

^§^These represent a combination of pre-XDR (n=5) and XDR (n=2).

**Figure 2:**
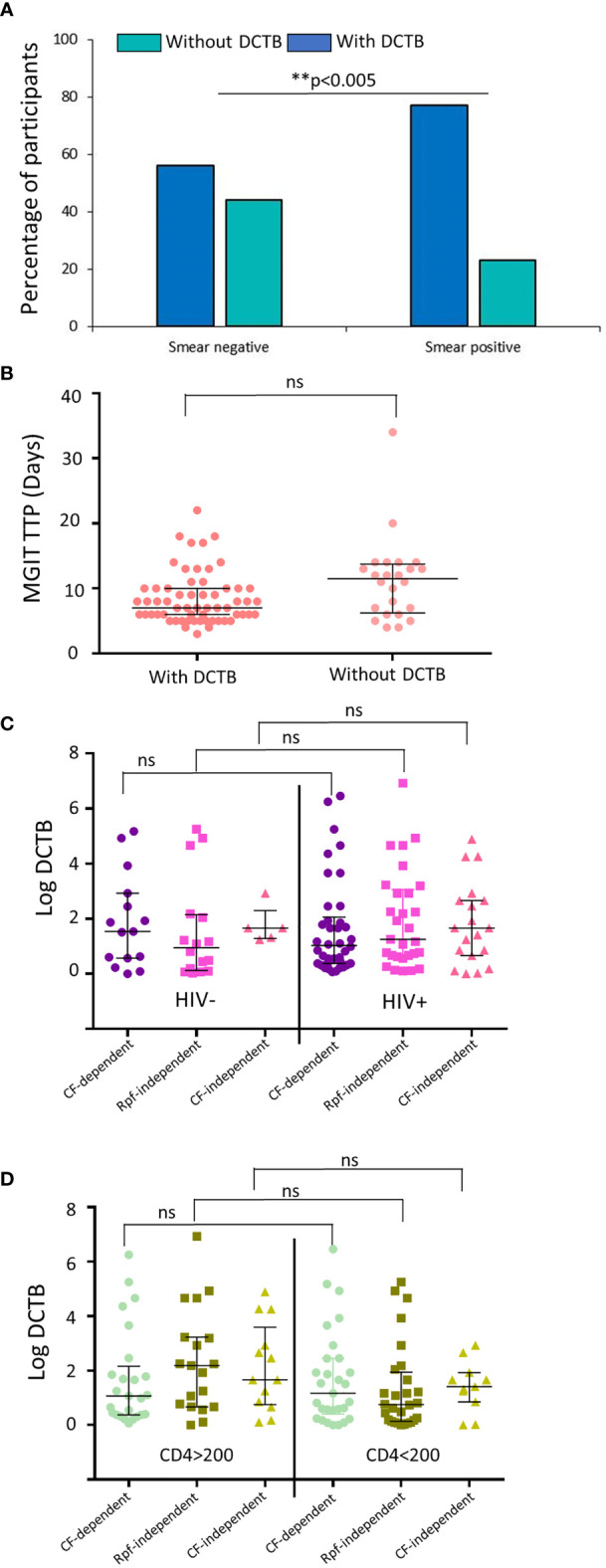
Prevalence of DCTB based on the laboratory diagnostics and HIV status of the participants. **(A)** Proportion of participants with (dark blue) and without (teal) DCTB based on their smear status. **(B)** Median mycobacterial growth indicator tube time to positivity (MGIT TTP) in specimens with and without DCTB. **(C)** Median DCTB (MPN/CFU) counts in specimens from participants stratified by HIV status. **(D)** Median DCTB (MPN/CFU) counts in specimens from HIV-infected participants stratified by CD4 counts. Values from specimens that had detectable levels of DCTB were used. Error bars depict the interquartile range. ** p-value <0.005 and ns, not significant.

Next, we assessed if the smear status of participants influenced the detection of DCTB. Smear-positive participants demonstrated the presence of DCTB in a significantly higher proportion (76%) of samples compared with smear-negative samples (56%) (**Figure 2A**). In contrast, there was no significant difference in MGIT time to positivity between samples with detectable levels of DCTB and those that had no DCTB (**Figure 2B**). In our previous work, we demonstrated that participants with HIV-TB coinfection harbored lower amounts of DCTB when compared with their HIV-uninfected counterparts (Chengalroyen et al., 2016). In contrast, the HIV status of participants with DR-TB did not affect the prevalence of DCTB as there was no significant difference in the amount of CF-dependent or Rpf-independent DCTB recovered between these groups (**Figure 2C**). Stratification of the participants based on CD4 counts showed no difference in the detection of DCTB (**Figure 2D**). In HIV-infected individuals, including those with CD4 counts <200 cells/mm^3^, supplementation with CF (with or without Rpfs) did have a significant effect in the detection of DCTB compared with un-supplemented MPN assays (**Supplementary Figures 2A, B**). There was no difference in MGIT time to positivity or CFU between the RMR, MDR, and XDR groups (in all cases, p>0.5). Similarly, when the participants were stratified based on their HIV status, there was also no difference in MGIT time to positivity or CFU between HIV-positive and HIV-negative participants (in all cases, p>0.5).

### Effects of drug treatment on the detection of DCTB

**Figure 3:**
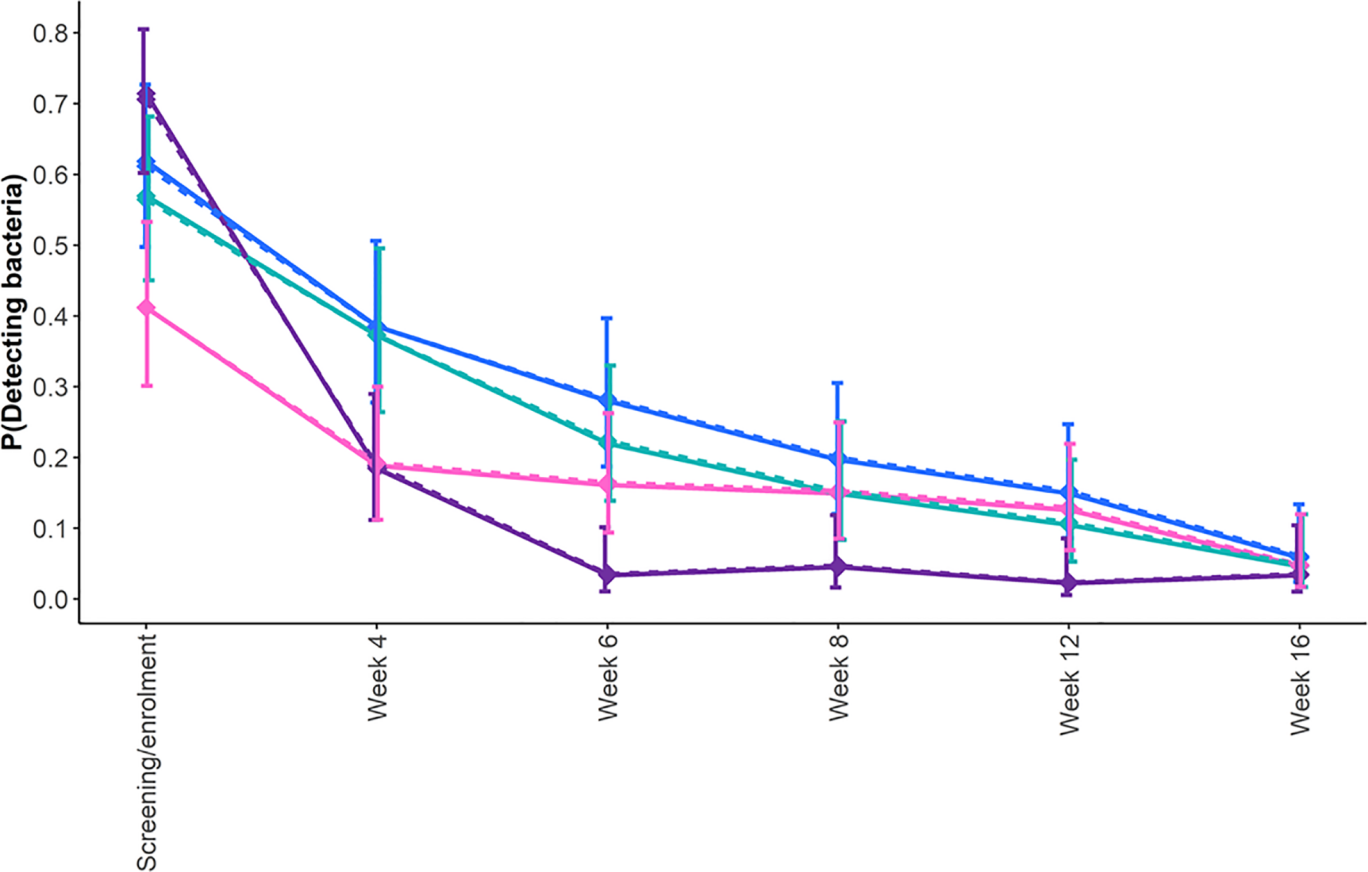
Estimated probability of detecting bacteria longitudinally in sputum specimens from participants with drug-resistant TB during treatment. Shown are the profiles of the probability of detecting DCTB within any of the MPN assays, CF-dependent DCTB (blue line); Rpf-independent DCTB (pink line); and CF-independent DCTB (teal line) and CFU (purple line) from screening/enrollment to 12 weeks after randomization. To determine the differences between the probabilities of detecting bacteria at each time point and across time points, we fitted a longitudinal generalized estimating equation (the results are presented in **Table S2**). The solid lines represent the adjusted model, and the dotted lines represent the unadjusted model.

During treatment with second-line drugs, the disposition of the cohort changed as two participants died after 5 and 16 months of therapy, two others refused further participation after 3 and 6 months, respectively, while an additional two were terminated at months 5 and 9 as per the investigator’s decision. The longitudinal DCTB data from the remaining participants were modeled up to week 16 (4 months) as beyond this treatment time point, insufficient levels of DCTB were detected to fit the model. The estimated probabilities from the unadjusted and adjusted model showed that the probability of detecting all forms of DCTB (CF-dependent, Rpf-independent, and CF-independent) reduced over time (**Figure 3**). On average, across all time points, using CF-dependent DCTB as reference, the odds of detecting bacteria reduced by 70%, 40%, and 20% for CFU, Rpf-independent DCTB, and CF-independent DCTB, respectively (**Figure 3**; **Table S2**).

At screening/enrolment, there was a 50% increase in the odds of bacterial detection by the CFU assay, while there was a 20% and 55% reduction for CF-independent and Rpf-independent DCTB, respectively, compared with CF-dependent DCTB (**Figure 3**; **Table S2**). However, 4 weeks after screening, there was a 63%, 5%, and 62% decrease in the odds of detecting CFU, CF-independent DCTB, and Rpf-independent DCTB, respectively, compared with CF-dependent DCTB. Six weeks post-treatment initiation, these odds were 90%, 30%, and 50% lower, respectively. While these odds remained lower than the CF-dependent DCTB at 8, 12, and 14 weeks post-treatment, the CFU comparisons showed statistical significance at weeks 8 and 12 (confidence intervals shown in **Table S2**). Smear-positive, XDR, and HIV-positive participants had 90%, 60%, and 60% higher odds of detecting bacteria, respectively, when compared with their counterpart groups; this difference was statistically significant (**Table S2**). There was no evidence of age, gender, and BMI influencing the detection of bacteria (**Table S2**). The inclusion of covariates in the model did not affect the effect of the CFs and their interaction with time.

The second-line drug regimen for 81% of the participants regardless of resistance profile consisted mainly of a combination of isoniazid, pyrizanamide, ethambutol, levofloxacin, ethionamide, clofazimine, bedaquiline, and linezolid (**Table S3**). This suggested that the inclusion of bedaquiline and linezolid, new or repurposed drugs, respectively, facilitated the clearance of both conventionally culturable and DCTB bacterial populations in individuals infected with DR-TB. However, in 19% of the participants, bedaquiline and linezolid were not administered and replaced primarily with kanamycin and moxifloxacin, respectively. Hence, we evaluated if treatment regimens lacking these drugs cleared DCTB after 4 weeks of treatment. Our analysis shows that the proportion of DCTB detected in participants on regimens without bedaquiline and linezolid was not significantly different compared with those whose regimens included these drugs (p= 0.3634, Fisher’s exact test).

## Discussion

Improving the efficiency of culture-based diagnostic tests to detect all organisms in sputum, such as DCTB, is imperative to enhance linkage to care and assess effectiveness of novel regimens for treatment shortening. Given that novel TB treatments should be active on currently circulating DR *M. tuberculosis* strains, we investigated the prevalence of DCTB in South African participants with DR-TB and also assessed the kinetics of bacterial clearance. In this work, DCTB populations were detected for a large proportion of participants infected with RMR, MDR, and XDR strains; however, it should be noted that the small sample size for XDR-TB limits definitive conclusions. We found that specimens containing MDR isolates displayed higher levels of Rpf-independent and CF-independent DCTB when compared with RMR isolates. Prior work demonstrated that a rifampicin-resistant isolate was limited in its ability to form DCTB during exposure to rifampicin, a phenomenon that was ascribed to the inability to inactivate the drug target (Saito et al., 2017). Considering this, the increased prevalence of DCTB in MDR isolates may be related to the inclusion of isoniazid resistance conferring mutations, where differences in mutational spectrum and drug modes of action could affect the establishment of DCTB populations. However, this observation requires further study for definitive conclusions.

Exclusion of Rpfs from the CF yielded more DCTB in specimens from participants with MDR-TB. As these molecules have been implicated in growth stimulation, this observation was counterintuitive. An inhibitory effect of CF supplementation in sputum culture assays has been noted in previous studies (Mukamolova et al., 2010; McIvor et al., 2021); however, whether this effect was specific to Rpfs in CF was not investigated. In addition, our prior work demonstrated that exclusion of Rpfs did not notably affect growth stimulatory capacity of CF derived from *Mycobacterium smegmatis*, suggesting that a combination of factors, specific to *M. tuberculosis*, are responsible for enhancing DCTB recovery (Gordhan et al., 2021). It is possible that MDR strains differ significantly in their capacity to detect growth stimulatory molecules or have inherent differences in growth that affect their recovery in MPN assays. Indeed, experiments in *ex vivo* models demonstrate that different MDR isolates have varying capacities to colonize macrophages and induce cytokines, which the authors ascribe to differences in growth rates (Yokobori et al., 2013).

Host immunity, together with drug treatment, is thought to drive the formation of, or select for, drug-tolerant populations during TB pathogenesis. In prior work with specimens from treatment-naïve individuals with drug-susceptible TB, comparison of the prevalence of DCTB in immunocompromised individuals (as defined by HIV infection and low CD4 counts) confirmed that a functional host immune response is associated with higher levels of DCTB (Chengalroyen et al., 2016). However, in our DR cohort, there was no difference in the amount of detectable DCTB between HIV-seropositive or -negative participants. In contrast to our findings with drug-susceptible TB, participants with CD4 counts of <200 cells/mm^3^ showed a significant increase in the detection of Rpf-independent DCTB. This suggests that immunometabolism in individuals with MDR-TB could be distinct from drug-susceptible TB. Consistent with this, it has been demonstrated that MDR W-strains of *M. tuberculosis* overexpress cell wall lipids that facilitate bypasses of the interleukin 1 receptor type I (IL-1R1) signaling pathway, leading to reprogramming of macrophage metabolism (Howard et al., 2018). These data suggest that MDR isolates may be exposed to a distinct intracellular host environment that can affect the prevalence of DCTB in sputum, an effect that will most likely be influenced by HIV-infection status. Targeting the use of DCTB assays in these individuals may provide programmatic benefit in the diagnostic pickup of TB infection.

Patients treated with bedaquiline containing regimens have a 65%–100% culture conversion rate with reasonable treatment outcomes, and combination with linezolid is proposed to increase the effectiveness of bedaquiline (Li et al., 2019). The treatment regimen for 81% of the patients in our cohort contained bedaquiline and linezolid. Longitudinal assessment for growth during treatment revealed that most patients cleared all conventionally culturable bacteria by 2 weeks of treatment, as observed by the steep decline in the CFUs. However, the estimated probability of detecting DCTB after 4 weeks of treatment was high under all liquid culturing conditions. The magnitude of the estimated effect of CF on the probability of detecting bacteria was the highest with the CF-dependent supplement compared with the Rpf-independent, CF-independent, and CFU assessments. However, when measured by DCTB (MPN/CFU) assays, most of our participants had cleared DCTB after 3 months of therapy. These observations are consistent with previous reports in which DCTB in drug-susceptible and DR-TB were shown not to persist beyond 2–3 months of therapy (Almeida Junior et al., 2020; Zainabadi et al., 2021). However, the number of subjects used in these studies was small, and the treatment follow-up times were short, which limited a definitive longitudinal assessment. Based on our findings, it is clear that culture conversion for DR strains using routine tests, if negative after 2 months, should be interpreted with caution as the probability of the presence of non-culturable bacteria is still relatively high.

Collectively, our data highlight important differences in the prevalence of DCTB in individuals harboring rifampicin-resistant TB, with MDR isolates displaying a higher propensity to adopt the DCTB state in sputum. Detection of these appeared to be facilitated by use of CF devoid of Rpfs. Use of Rpf-deficient CF also enhanced the quantity of DCTB recovered in HIV-infected individuals with low CD4 counts. Detection of DCTB emerges as a promising new clinical endpoint to assess the durability of current cure readouts. These and other effects should be investigated in larger studies in programmatic settings.

## Materials and methods

All methods were performed in accordance with the relevant guidelines and regulations for growth of *M. tuberculosis* and handling of human specimens. All procedures were conducted in a BioSafety Level III laboratory, registered with the South African Department of Agriculture Forestry and Fisheries (registration number: 39.2/NHLS-20/010). All procedures were approved by the Institutional BioSafety Committee of the University of the Witwatersrand (approval number: 20200502Lab).

### Bacterial strains and culture conditions

The laboratory strain *M. tuberculosis* H37Rv and the corresponding mutant lacking all five *rpf* genes (Kana et al., 2008) was grown as previously described for the generation of culture filtrate (Chengalroyen et al., 2016).

### Recruitment of participants to obtain sputum specimens

A randomized controlled clinical trial comparing the treatment success of a gene-derived individualized drug-resistant TB regimen with a standard TB regimen (INDEX study) based on South African National Tuberculosis guidelines was set up at CAPRISA, UKZN, South Africa. Ethics approval for participant recruitment was obtained from the UKZN Biomedical Research Ethics Committee (UKZN BREC), South Africa, with clearance number BFC 584/16. Individuals 18 years and older attending provincial satellite sites with drug-resistant TB testing facilities in the greater Durban area, South Africa, were approached for participation into the study and enrolled between 10 March 2018 and 4 March 2021. Participants were allowed not more than 20 days of treatment initiation before screening/enrolment. HIV-seronegative and -positive patients with drug-resistant TB, as determined by GeneXpert, were eligible for enrolment in the study. HIV-positive patients already on antiretroviral were also included in the study. Only patients with line probe assay (LPA) results consistent with MDR-TB, pre-XDR, and/or XDR-TB were included in the study. Patients willing to participate were approached, and written informed consent was obtained using Informed Consent Forms reviewed and approved by the UKZN, BREC. Patients were followed up for 25 months at various times during treatment. After written informed consent was granted, an additional spot sputum was collected for analysis by the most probable number (MPN) assay (otherwise referred to as limiting dilution assays) as described previously (Chengalroyen et al., 2016). The MPN assay is a limiting dilution series based on a Poisson distribution for the quantification of bacterial growth in liquid media, described in further detail in Supplementary Information. Sputum samples were serially diluted in a 48-well microtiter plate in media with culture filtrate (1:1 ratio) purified from wild-type *M. tuberculosis*. In addition, select dilutions of the sputum were spread on solid 7H11 plates to determine the CFUs. These assays are described in detail in Supplementary Information.

## Data analysis

Statistical analysis was done using GraphPad Prism software, version 6; R software 4.2.1; and SAS 9.4. For assessing the effect of different CFs (and the no CF control) to recover DCTB, paired t-tests were used. All samples (including those that did not give DCTB with either CFs or in the no CF assay) were used for these comparisons. When comparing the quantity of DCTB recovered between categories (resistance categories, HIV status, or CD4 counts), unpaired t-tests were used and data from all specimens yielding no DCTB were excluded. For comparing proportions of individuals positive for DCTB and CFU during treatment, Fisher’s exact test was used. The comparisons of the supplement effect were done with reference to the effect of the CF-dependent as it was anticipated to be more effective than the CF-independent, Rpf-independent, and the CFU. Longitudinal data were subjected to a generalized linear model that accounts for the repeated measurements per participant due to multiple time points and different CF supplementation to the probability of detecting bacteria. The parameters were estimated using a generalized estimating equations that assumed exchangeable working correlation. The time was treated as a categorical covariate. We further extended the model by adjusting for baseline covariates, thus adding the effect of gender (men or women), age (in years), HIV status (negative or positive), drug resistance (RMR, MDR, or XDR), and smear (negative or positive).

## Data availability statement

The original contributions presented in the study are included in the article/**Supplementary Material**. Further inquiries can be directed to the corresponding author.

## Ethics statement

The studies involving human participants were reviewed and approved by University of Kwazulu Natal ethics number BFC 584/16. The patients/participants provided their written informed consent to participate in this study.

## Author contributions

BK conceived the overall concept of the study. BG and AS executed the laboratory aspects of the study. NP, KN, NYZ and TC recruited the study participants. BG and BK wrote the first draft of the manuscript. ML assisted with data analysis. All authors contributed to the article and approved the submitted version.

## Funding

The primary study was supported by funding from EDCTP, grant TMA2018SF to CAPRISA. The laboratory analysis of the specimens was supported by funding from the South African National Research Foundation (to BK), the South African Medical Research Council (to BK), and the National Health Laboratory Service Research Trust (to BG).

## Acknowledgments

We thank CAPRISA for recruiting participants and collecting and transporting the sputum samples. We are thankful and grateful to the participants for providing their valuable specimens for this study.

## Conflict of interest

The authors declare that the research was conducted in the absence of any commercial or financial relationships that could be construed as a potential conflict of interest.

## Publisher’s note

All claims expressed in this article are solely those of the authors and do not necessarily represent those of their affiliated organizations, or those of the publisher, the editors and the reviewers. Any product that may be evaluated in this article, or claim that may be made by its manufacturer, is not guaranteed or endorsed by the publisher.
