# Supplementary material for: Detection of differentially culturable tubercle bacteria in sputum from drug-resistant tuberculosis patients
Source: Front Cell Infect Microbiol. 2022 Sep 9;12:949370. doi: 10.3389/fcimb.2022.949370 (PMC9500503; doi:10.3389/fcimb.2022.949370)
Supplement: Supplementary file 1 [file DataSheet_1.pdf]

Supplementary Methods

Recruitment criteria

For inclusion in the study, participants had to be ≥18 years with pulmonary TB, that are Xpert MTB/RIF assay positive with resistance to rifampicin. The participants had to have LPA results consistent with MDR-TB, pre-XDR and/or XDR-TB. Both HIV-uninfected and infected individuals were recruited to the study including those on ARVs. The participants had to be willing to provide an address or mobile phone number where they could be contacted and willing to have samples collected, stored indefinitely and used for research purposes. Individuals suffering from any acute condition or any other chronic or clinically significant medical condition were excluded. All patient names were anonymised and given a unique study identity. Three spot sputum samples were collected at CAPRISA and shipped on that day to the National Institute for Communicable Disease (NICD). The temperature of the sputum samples was monitored with a thermometer during shipment from CAPRISA to the NICD as the samples were transported by air and were in transit for approximately 5-6 hours. Once the package was received at the NICD the relevant samples for testing at the NICD together with the thermometer was removed. The samples for the CBTBR were batched and placed at 4°C until shipment twice a week to the CBTBR. In this case, the samples were transported over a short distance (14 km), frozen ice packs were placed in the box during transport. Upon arrival at the CBTBR, the samples were placed at 4°C. All samples were processed within 1 week of receipt from CAPRISA.

Testing of sputum samples

Multiple spot sputum samples were collected for the various arms of the parent study. One sample was used to carry out the routine diagnostic tests for TB detection (Smear, MGIT, LPA), the second sample was analysed at the NICD for whole genome sequencing and the third sample was used for the DCTB assay. All baseline and follow up samples were batched and processed in real time within a week to assess for CFU and MPN

Decontamination of sputum samples

A 1:1 volume of 2.9% sodium citrate and 4% sodium hydroxide was added to the sputum sample and incubated at room temperature for 20 min followed by centrifugation at 3900xg for 10 min. The cell pellet was washed with 45 ml of 0.01 M phosphate buffered saline (PBS), pH 7.4. For all sputum samples regardless of the collection volume, the pellet was resuspended in 2 ml of Middlebrook 7H9 media supplemented with 10% OADC, 0.2% glycerol and 0.05% Tween80 (7H9). Half this volume was used for the Most Probable Number (MPN) analysis and CFU assessment and the remainder was frozen at -80°C.

Bacterial culturing and culture filtrate (CF) preparation procedures

Cultures of *M. tuberculosis* H37Rv were grown by inoculating 1 ml freezer stocks (OD_{600nm} 0.5-0.8) into 8 ml of 7H9 and grown for 2.5 days to an OD_{600nm} of ~0.5. The pre-culture was added to 42 ml of 7H9 media and allowed to grow for 2-3 days to an OD_{600nm} of 0.6-0.8. CF was obtained by centrifugation of the cultures for 10 min at 3900x g and filtration of the supernatants through a 0.22 µM filter attached to a 50 ml syringe. CF from *Mtb* was prepared in real time for each batch of sputum samples. The filtered CF was diluted with 50% 7H9 media and supplemented with 8% (w/v) PANTA (polymyxin, amphotericin B, naladixic acid, trimethoprim and azlocillin, Becton Dickinson, South Africa) to manage contamination.

To confirm sterility of the CF, 1 ml aliquots of each was incubated at 37 °C for 3 months with an additional aliquot spread on 7H11 media and the plates incubated at 37 °C for 8 weeks. Aliquots of

the CF were also screened by PCR for select *rpf* genes using *Mtb rpf* gene specific primers (Table S1) (Kana et al., 2008) to confirm the strain genotype and to ensure no cross contamination of strains occurred during CF preparation.

The MPN assay

The MPN assay was performed as previously described (Mukamolova et al., 2010; Chengalroyen et al., 2016). Briefly, 450 µl of each of the PANTA supplemented CFs were dispended across 3 columns (triplicate) in all wells in a 48 well microtitre plate (Figure 1B). Media supplemented with PANTA was used as the control in triplicate in the remaining three columns. The 1 ml decontaminated sputum sample was transferred to a 50 ml Falcon tube containing 0.5 ml of sterile 2 mm glass beads and vortexed for about 30 seconds to break up bacterial clumps/cords. Fifty microlitres of the sputum sample was added to all the wells in the first row and 10 fold dilutions were carried out across the plate until the last row from which 50 µl of sample was discarded (Figure 1B). The plates were sealed with biohazard tape and incubated at 37 °C for six weeks, followed by visual scoring of growth using an inverted mirror. The total number of bacteria in the MPN assay was estimated using software available at http://www.wiwiss.fu.berlin.de/fachbereich/vwl/iso/ehemalige/wilrich/index.html.

Determination of colony forming units (CFUs)

The CFU's were determined by spreading appropriate 10 fold dilutions of the decontaminated sputum onto Middlebrook 7H11 media supplemented with 0.5% glycerol and 10% OADC (Figure 1B). The plates were incubated at 37 °C for four weeks before scoring colonies.

Supplementary Figures

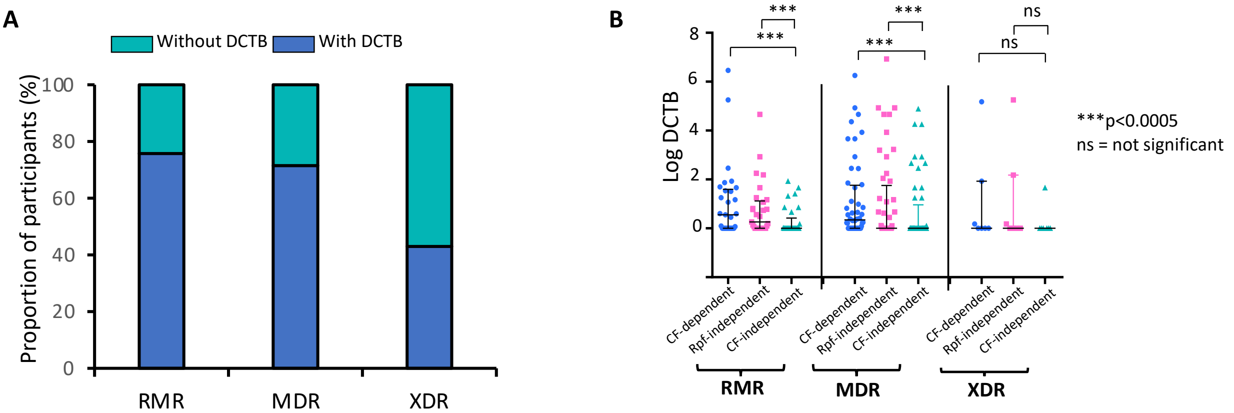

Figure S1. DCTB analysis in baseline sputum samples. (A) The proportion of participants with (dark blue) and without (teal) DCTB for patients stratified as rif–mono resistant (RMR), multidrug resistant (MDR) and extremely drug resistant (XDR) TB. **(B)** Dot plot depicting median DCTB counts from different MPN assays and resistance categories. To compare the effect of CF/Rpfs in growth stimulation, a paired t-test was used within each drug resistance category. Error bars depict the interquartile range.

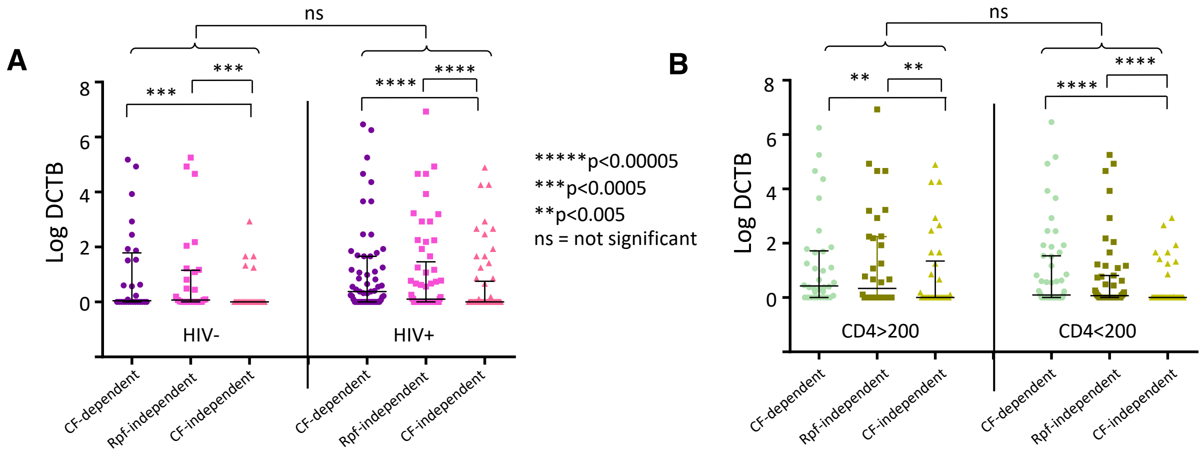

Figure S2. DCTB prevalence in participants with HIV infection. (A) Median DCTB counts in specimens from participants stratified by HIV status. (B) Median DCTB counts in specimens from participants stratified by CD4 counts. To compare the effect of CF/Rpfs in growth stimulation, a paired t-test was used. Error bars depict the interquartile range.

Supplementary Tables

Table S1: List of PCR primers used in this study

Gene	*Primer Name*	*Primer Sequence (5' – 3')*
Rv0867c (*rpfA[Mtb]*)	Mtb_rpfA-F<br>Mtb_rpfA-R	CGGGTTATCGAACGCAACAC<br>GGTCGTTAGCGGCAAGTTCC
Rv1884c (*rpfC[Mtb]*)	Mtb_rpfC-F<br>Mtb_rpfC-R	AGCTGCCTCTCGGGAACAAC<br>GACCACAGTGCGATCGGAAG

Table S2: Estimated odds ratio and adjusted odd ratio from the generalised estimates equation that predicted the probability of detecting DCTB

Effect	Time point	Difference [(a)]	Unadjusted	Adjusted
CF		CF-independent DCTB (CF-dependent DCTB)	0.777 (0.461,1.307)	0.770 (0.459,1.290)
		CFU (CF-dependent DCTB)	**0.304 (0.144,0.640)	**0.299 (0.142,0.632)
		Rpf-independent DCTB (CF-dependent DCTB)	*0.584 (0.355,0.960)	*0.577 (0.351,0.948)
Time		Week 4 (Screening/enrolment)	***0.278 (0.183,0.423)	***0.269 (0.175,0.413)
		Week 6 (Screening/enrolment)	***0.124 (0.072,0.214)	***0.118 (0.068,0.205)
		Week 8 (Screening/enrolment)	***0.104 (0.061,0.179)	***0.099 (0.057,0.171)
		Week 12 (Screening/enrolment)	***0.069 (0.036,0.134)	***0.065 (0.033,0.128)
		Week 16 (Screening/enrolment)	***0.037 (0.018,0.074)	***0.034 (0.017,0.070)
CF-dependent DCTB compared by Time	Screening/ enrolment	CF-independent DCTB (CF-dependent DCTB)	0.823 (0.395,1.714)	0.817 (0.392,1.700)
		CFU (CF-dependent DCTB)	1.523 (0.708,3.276)	1.540 (0.713,3.326)
		Rpf-independent DCTB (CF-dependent DCTB)	*0.444 (0.213,0.927)	*0.432 (0.206,0.909)
	Week 4 (randomization)	CF-independent DCTB (CF-dependent DCTB)	0.951 (0.447,2.024)	0.947 (0.437,2.049)
		CFU (CF-dependent DCTB)	*0.371 (0.159,0.862)	*0.361 (0.152,0.857)
		Rpf-independent DCTB (CF-dependent DCTB)	*0.380 (0.163,0.886)	*0.371 (0.155,0.888)
	Week 6	CF-independent DCTB (CF-dependent DCTB)	0.732 (0.315,1.698)	0.724 (0.314,1.666)
		CFU (CF-dependent DCTB)	**0.093 (0.021,0.420)	**0.090 (0.020,0.408)
		Rpf-independent DCTB (CF-dependent DCTB)	0.501 (0.204,1.231)	0.494 (0.201,1.213)
	Week 8	CF-independent DCTB (CF-dependent DCTB)	0.722 (0.276,1.886)	0.715 (0.267,1.916)
		CFU (CF-dependent DCTB)	*0.198 (0.050,0.779)	*0.194 (0.048,0.776)
		Rpf-independent DCTB (CF-dependent DCTB)	0.722 (0.276,1.886)	0.718 (0.268,1.922)

Effect	Time point	Difference (a)	Unadjusted	Adjusted
	Week12	CF-independent DCTB (CF-dependent DCTB)	0.672 (0.225,2.009)	0.665 (0.219,2.020)
		CFU (CF-dependent DCTB)	*0.133 (0.021,0.841)	*0.131 (0.021,0.836)
		Rpf-independent DCTB (CF-dependent DCTB)	0.823 (0.289,2.348)	0.821 (0.284,2.377)
	Week 16	CF-independent DCTB (CF-dependent DCTB)	0.788 (0.159,3.914)	0.781 (0.154,3.951)
		CFU (CF-dependent DCTB)	0.567 (0.098,3.275)	0.565 (0.096,3.325)
		Rpf-independent DCTB (CF-dependent DCTB)	0.790 (0.159,3.917)	0.791 (0.158,3.952)
Smear Results		Positive (Negative)		**1.882 (1.329,2.666)
Drug resistance		XDR (MDR)		*1.602 (1.003,2.558)
HIV status		Positive (Negative)		*1.559 (1.180,2.060)
Gender		Female (Male)		1.225 (0.906,1.658)
Age				1.011 (0.995,1.027)
BMI				0.992 (0.943,1.044)

[a] reference for comparison in parentheses, for example, CFU (CF-dependent) – CFU is compared to CF-Dependent DCTB as a reference
* p-value <0.05; ** p-value <0.001; * p-value <0.0001

Table S3: Tuberculosis drug regimen for participants infected with RMR, MDR or XDR strains

DR No	PID	Drug resistance	DCTB	INH	PZA	ETB	LVF	ETN	CFZ	BDQ	LNZ	KAN	MXF	APM / CVN	IMP / CLS	DLM	PAC	RIF	RIB	CPM	TZD	GFX
54	020-10-0170	RMR	Y																			
24	020-10-0140	RMR	N																			
34	020-10-0151	RMR	N																			
55	020-10-0169	RMR	Y																			
83	020-10-0201	RMR	Y																			
122	020-10-0243	RMR	N																			
125	020-10-0247	RMR	Y																			
48	020-10-0165	RMR	Y																			
70	020-10-0185	RMR	Y																			
87	020-10-0203	RMR	N																			
76	020-10-0194	RMR	Y																			
120	020-10-0242	RMR	N																			
17	020-10-0133	RMR	Y																			
39	020-10-0157	RMR	Y																			
22	020-10-0138	RMR	Y																			
96	020-10 0213	RMR	Y																			
89	020-10-0205	RMR	Y																			
102	020-10-0219	RMR	Y																			
58	020-10-0178	RMR	Y																			
51	020-10-0171	RMR	Y																			
21	020-10-0137	RMR	Y																			
135	020-15-0257	RMR	Y																			
67	020-10-0183	RMR	Y																			
52	020-10-0177	RMR	Y																			
124	020-10-0246	RMR	Y																			
57	020-10-0172	MDR	Y																			
103	020-10-0220	MDR	Y																			
101	020-10-0218	MDR	Y																			
121	020-10-0244	MDR	Y																			
78	020-10-0195	MDR	Y																			
36	020-10-0153	MDR	Y																			
117	020-10-0239	MDR	Y																			
105	020-10-0221	MDR	N																			
107	020-10-0224	MDR	Y																			
98	020-10-0216	MDR	Y																			
79	020-10-0193	MDR	N																			
23	020-10-0139	MDR	Y																			
42	020-10-0161	MDR	Y																			
31	020-10-0147	MDR	N																			
44	020-10-0162	MDR	Y																			
99	020-10-0215	MDR	Y																			
136	020-10-0255	MDR	Y																			
134	020-10-0256	MDR	Y																			
130	020-10-0251	MDR	Y																			
93	020-10-0211	MDR	Y																			
26	020-10-0142	MDR	N																			
18	020-10-0134	MDR	Y																			
82	020-10-0199	MDR	Y																			
45	020-10-0163	MDR	Y																			
49	020-10-0164	MDR	Y																			
66	020-10-0182	MDR	Y																			
85	020-10-0202	MDR	Y																			
35	020-10-0152	MDR	Y																			
14	020-10-0130	MDR	N																			
88	020-10-0206	MDR	Y																			
65	020-10-0180	MDR	Y																			
40	020-10-0158	MDR	Y																			
92	020-10-0209	MDR	Y																			
91	020-10-0208	MDR	N																			
81	020-10-0198	MDR	Y																			
64	020-10-0181	MDR	Y																			
131	020-10-0252	MDR	Y																			
60	020-10-0175	MDR	Y																			
30	020-10-0146	XDR	N																			
127	020-10-0249	XDR	Y																			
37	020-10-0155	XDR	Y																			
27	020-10-0143	XDR	Y																			
86	020-10-0204	XDR	N																			

RMR: rifampicin mono resistant (in blue), MDR: multi drug resistant (in green) and XDR: extensively drug resistance (in purple). Y = yes; N = no
INH: isoniazid; PZA: pyrazinamide; ETB: ethambutol; LVF: levofloxacin; ETN: ethionamide; CFZ: clofazimine; BDQ: bedaquiline; LNZ: linezolid; KAN: kanamycin; MXF: moxifloxacin; APM/ CVN: amoxicillin/clavulanate; IMP / CLS: imipenim/cilastatin; DLM: delaminid;; PAC: para aminosalicyclic acid; RIF: rifampicin; RIB: rifabutin; CPM: capreomycin; TZD: terizidone; GFX: gatifloxacin.

References

Chengalroyen, M.D., Beukes, G.M., Gordhan, B.G., Streicher, E.M., Churchyard, G., Hafner, R., Warren, R., Otwombe, K., Martinson, N. and Kana, B.D. (2016). Detection and Quantification of Differentially Culturable Tubercle Bacteria in Sputum from Patients with Tuberculosis. *Am J Respir Crit Care Med* 194**,** 1532-1540. 10.1164/rccm.201604-0769OC

Kana, B.D., Gordhan, B.G., Downing, K.J., Sung, N., Vostroktunova, G., Machowski, E.E., Tsenova, L., Young, M., Kaprelyants, A., Kaplan, G. and Mizrahi, V. (2008). The resuscitation-promoting factors of *Mycobacterium tuberculosis* are required for virulence and resuscitation from dormancy but are collectively dispensable for growth in vitro. *Mol Microbiol* 67**,** 672-684. 10.1111/j.1365-2958.2007.06078.x

Mukamolova, G.V., Turapov, O., Malkin, J., Woltmann, G. and Barer, M.R. (2010). Resuscitation-promoting factors reveal an occult population of tubercle bacilli in sputum. *Am J Respir Crit Care Med* 181**,** 174-180. 10.1164/rccm.200905-0661OC
